# Cultural Differences in the Perception of Daily Stress Between European Canadian and Japanese Undergraduate Students

**DOI:** 10.1177/01461672211070360

**Published:** 2022-02-25

**Authors:** Hajin Lee, Takahiko Masuda, Keiko Ishii, Yuto Yasuda, Yohsuke Ohtsubo

**Affiliations:** 1 University of Montréal, Quebec, Canada; 2 University of Alberta, Edmonton, Canada; 3 Nagoya University, Aichi, Japan; 4 The University of Tokyo, Bunkyo-ku, Japan

**Keywords:** culture, daily stress, situation sampling, social orientation

## Abstract

The current research examines cross-cultural differences in people’s daily stress experiences and the role of social orientations in explaining their experiences. Using a situation sampling method, Study 1 collected European Canadian and Japanese undergraduates’ examples of stressful interpersonal and non-interpersonal situations they experienced, measuring participants’ perception of the intensity and frequency of each type of situation. Studies 2 and 3 examined the effects of culture on participants’ reports of stress symptoms under the situations. Study 3 assessed the mediating effects of independence and interdependence between culture and perceived stress. These studies indicated that the situational context moderates the effect of culture on perceptions of stress, showing a different amount of stress from interpersonal situations between Japanese and European Canadian undergraduates. Mediational analyses revealed that independent orientation partially explains the relationship between culture and stress from interpersonal situations. The implications of these results for culture and daily stress are discussed.

*Stress* entails psychological or physical reactions to a challenging situation. People all over the world experience stress on a daily basis and these experiences significantly affect their well-being (Bolger et al., 1989). In North American community health surveys, 20% of Canadians perceived most days in their life as “quite a bit” or “extremely” stressful (Statistics Canada, 2017) and 75% of Americans experienced at least one symptom of stress in the past month (American Psychological Association, 2015). While stress can be positive or negative, *distress* is characterized an adverse stress response, often involving negative affect and physiological reactivity. When people experience frequent and intense day-to-day stress, they may also experience distress. The cumulative impact of distress from daily stress plays a role in mental and physical illnesses (Charles et al., 2013; Leger et al., 2018), and even brings a higher risk of mortality (Chiang et al., 2018).

Considering the impact of daily stress on well-being and health, it is important to understand what situations people perceive as stressful. Daily stressful situations encompass a wide range of situations that vary in intensity and frequency, for example, being stuck in traffic, having an argument with a spouse, or facing a deadline at work. Here, we focus on two types of situations: *interpersonal situations* (involving interactions with others), and *non-interpersonal situations* (not involving interactions with others).

Cultural psychologists have suggested that people’s perceptions of situations are shaped by differences in their social orientations across and within cultural regions (Markus & Kitayama, 2010; Varnum et al., 2010). Individuals from North American cultures tend to perceive themselves as being separate from others (*independent social orientation*) and prioritize independent values and goals (such as personal accomplishments) when evaluating situations. Conversely, individuals from East Asian cultures tend to view themselves as being embedded in relationships with others (*interdependent social orientation*) and prioritize interdependent values and goals (such as social harmony) when evaluating situations. Similarly, these patterns of social orientation are reflected in attention patterns where North Americans mainly fixate on describing a person’s emotion rather than the surrounding social situation, whereas East Asians holistically consider relationships between a person and the social situation (Masuda et al., 2019).

In this article, we investigated how social orientations, shaped by cultural contexts, influence the daily stress experiences and perceptions of nonclinical undergraduate samples in Canada and Japan. We present three studies that adopted a situation sampling method (Kitayama et al., 1997) to 1) collect concrete examples of European Canadian and Japanese undergraduates’ interpersonal and non-interpersonal situations that involve daily stress, 2) assess how they perceive these situations, and 3) examine whether participants’ social orientations explain cultural differences in perceived stress elicited from these situations.

## Cultural Differences in Perception and Symptoms of Stressful Situations

Prior stress research targeting North Americans has separated stressful situations into two domains—interpersonal and non-interpersonal—and has shown that people’s perceived stress differs depending on the type of situation (Bolger et al., 1989). To date, few studies have focused on cultural differences in how people perceive different types of stressful situations in daily life. For example, Hashimoto and colleagues (2012) examined cultural differences in perceived frequency of stressful interpersonal situations (e.g., “I was insulted or ridiculed by others”) and their impact on psychological distress (e.g., feeling depressed or irritated), comparing American versus Japanese undergraduate samples. They found that compared with American undergraduates, Japanese undergraduates perceived stressful interpersonal situations as more frequent, which was associated with greater psychological distress. Although Hashimoto et al. (2012) exclusively focused on cultural differences in stressful interpersonal situations, our study builds on this work by comparing individuals’ perceptions of stressful interpersonal versus non-interpersonal situations.

Cross-cultural literature on well-being and health supports the claim that stronger independent social orientation is a protective factor against psychological distress (characterized by adverse stress responses), whereas stronger interdependent social orientation is a risk factor for vulnerability to distress (Okazaki, 2002). East Asian populations, who tended to score higher on interdependence and/or lower on independence, show greater vulnerability to psychological distress from social situations compared with North Americans, who tended to score higher on independence and/or lower on interdependence. Similarly, Kirmayer (1993) demonstrated that Japanese clinical populations often report greater psychological distress describing a wide range of stress from interpersonal relationships. The greater focus on social harmony in interdependently oriented cultures may create a higher sensitivity to interpersonal relationships, which may, in turn, predict greater psychological distress.

Prior research has also documented cultural variations in the symptoms of psychological distress that people focus on (Kirmayer & Ryder, 2016). While North Americans often emphasize psychological symptoms of distress, East Asians, including Chinese (Ryder et al., 2008), Koreans (Zhou et al., 2015), and Japanese (Kirmayer, 1993), often emphasize physical symptoms. Chentsova-Dutton et al. (2014) suggested that cultural differences in the focus of symptoms may reflect how people in different cultures have distinctive cultural scripts for their emotional experiences. North Americans tend to place greater value on recognizing and expressing their psychological states or emotions. Conversely, East Asians tend to have lay beliefs that psychological states and bodily sensations are mutually constitutive and place greater value on being emotionally reserved for the sake of social harmony.

Evidence supports cultural variations in the experience of psychological distress through two strands of research: one on an association of social orientations with psychological distress and the other on the focus of psychological versus physical symptoms of distress. Although prior studies have extensively documented cultural differences in the severity of psychological distress such as depression or anxiety (Chentsova-Dutton et al., 2014), the topic of cultural differences in daily stress has received relatively scant attention in the literature. Given that daily stress can be a risk factor for severe psychological distress (Charles et al., 2013), it is important to elucidate cultural influences in the extent to which people perceive daily stress in different types of situations and assess the role of social orientations in their perceptions.

## Current Research

The current research aims to understand daily stressful situations across cultures and examine cultural variations in perception of stressful situations. To achieve this, we used a situation sampling method (Kitayama et al., 1997) because it has the advantage of not only measuring how people from different cultures perceive their own situations but also analyzing how people evaluate culturally shared situations. In Study 1, we asked European Canadian and Japanese participants to concretely describe situations where they experienced stress from interpersonal situations and from non-interpersonal situations, and to rate each situation’s intensity and frequency. In Studies 2 and 3, we measured the extent to which participants would perceive stress psychologically or physically when they imagine themselves experiencing a random selection of the stressful situations generated in Study 1.

The current studies also assessed whether situational context moderates variation in European Canadian and Japanese undergraduates’ perceptions of daily stress, and the mediating effect of social orientations in explaining their perceptions of stress and well-being by testing the following questions: “Do European Canadians find stressful non-interpersonal situations more frequent and intense, and perceive more stress from non-interpersonal situations?”; “Do Japanese undergraduates find stressful interpersonal situations more frequent and intense, and perceive more stress from interpersonal situations?”; and “Does social orientation mediate the relationship between culture and stress from different types of situations?”

Under the rubric of the social orientation hypothesis (Varnum et al., 2010), we hypothesized that European Canadians would be more sensitive to stress in non-interpersonal situations and that Japanese people would be more sensitive to stress in interpersonal situations. We compared perceptions of interpersonal situations with non-interpersonal ones within each group and between the two groups (Studies 1 through 3). Given that prior studies showed the role of social orientation in the interaction between culture and psychological distress from social situations (Okazaki, 2002), we also expected that weaker independent and/or stronger interdependent orientation would predict greater stress, particularly from interpersonal situations (Study 3).

In addition, the current studies explored cultural differences in the symptoms of stress that people focus on. In line with cultural scripts for emotional experiences (Chentsova-Dutton et al., 2014), we predicted a greater focus on psychological symptoms among European Canadians and physical symptoms among Japanese participants (Study 1). We also predicted that these cultural differences in the focus of symptoms would appear even when participants were asked to imagine themselves in situations that others have experienced (Studies 2 and 3). We categorized the sources of stress described in the situation samples to better understand how stressful situations differ qualitatively across cultures (Supplemental Table S1) and included tables reporting the means, standard deviations, and correlations of the variables in each study (Supplemental Tables S2–S4). No studies in this manuscript were preregistered.

## Study 1

In this study, we examined how European Canadian and Japanese undergraduates perceive different types of daily stressful situations. We collected a similar number of stressful interpersonal and non-interpersonal situations from members of each culture, and assessed cultural variations in stress intensity level and the frequency of such situations. We expected higher ratings of frequency and intensity for interpersonal situations among Japanese undergraduates and for non-interpersonal situations among European Canadians. In addition, we analyzed the text of 160 situations to examine whether the use of words in the described situations reveals cultural differences in the focus of symptoms, predicting a greater focus on psychological symptoms of stress among European Canadians and physical symptoms among Japanese undergraduates when describing their situations.

### Method

#### Data availability

Materials and data for the three studies are openly available on OSF. https://osf.io/skder/

#### Participants

A priori power analysis using G*Power indicated that 94 participants would be sufficient to detect a two-way interaction with an effect size of $\eta_{p}^{2}$ = .08 and a power of .80 (Faul et al., 2009; Hashimoto et al., 2012). We recruited 104 participants, including 53 European Canadian undergraduates (73.6% female; *M*_age_ = 19.34, *SD* = 1.97, range = 18–28) at the University of Alberta in Canada and 51 Japanese undergraduates (52.9% female; *M*_age_ = 20.12, *SD* = 1.12, range = 18–23) at Kobe University in Japan. We excluded one Japanese participant from the analyses as the participant did not follow the instructions. The final sample consisted of 103 participants.

#### Measures and procedure

To collect situation samples from each culture, participants were asked to describe two situations in detail: one they have personally experienced in which they were stressed out in an *interpersonal context* and the other in which they were stressed out in a *non-interpersonal context* (related to their personal experiences). After writing down each situation, they were asked to rate two items, “How stressful was the situation you described above?” and “In general, how often do you experience situations in which you are stressed out in an interpersonal/non-interpersonal context?” on a scale from 1 (*not at all/never*) to 9 (*very much/always*). These measures were translated and back-translated by a team of bilingual Japanese–English researchers. Participants also provided demographic information, such as gender and age.^1^

### Results

#### Cultural differences in perceived stress intensity of situations

A 2 (Culture: European Canadian vs. Japanese; between-Ss) × 2 (Situation: interpersonal vs. non-interpersonal; within-Ss) mixed factorial analysis of variance (ANOVA) yielded a main effect of Situation, *F*(1, 101) = 5.69, *p* = .019, $\eta_{p}^{2}$ = .053, 90% confidence interval (CI) = [.005, .137],^2^ showing a higher level of stress intensity for non-interpersonal situations (*M* = 7.23, *SD* = 1.40) than interpersonal ones (*M* = 6.80, *SD* = 1.42). There was no significant main effect of culture, *F*(1, 101) = 0.02, *p* = .894, $\eta_{p}^{2}$ = .000 [.000, .008].

**Figure 1.**
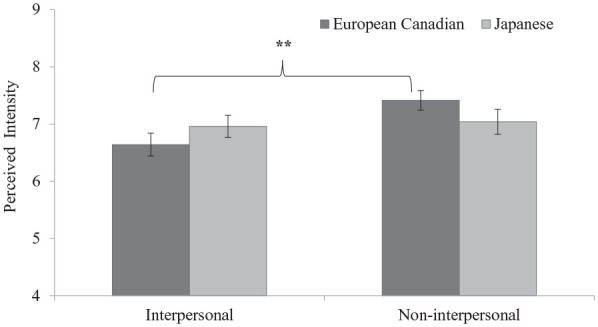
Cultural differences in perceived stress intensity of situations in Study 1. *Note.* Error bars depict standard errors. **p < .01 (two-tailed).

The main effect of situation was qualified by an interaction with culture, which approached significance, *F*(1, 101) = 3.75, *p* = .055, $\eta_{p}^{2}$ = .036 [.000, .111]. Simple-effects analysis indicated that there was a nonsignificant difference between the two groups in perceived stress intensity of interpersonal situations (European Canadians: *M* = 6.64, *SD* = 1.46; Japanese: *M* = 6.96, *SD* = 1.37), *t*(101) = −1.15, *p* = .253, *d* = 0.227, 95% CI = [−0.162, 0.614], and non-interpersonal situations (European Canadians: *M* = 7.42, *SD* = 1.25; Japanese: *M* = 7.04, *SD* = 1.54), *t*(101) = 1.35, *p* = .179, *d* = 0.267, 95% CI = [−0.122, 0.654]. However, paired *t* tests for each culture showed that the perceived stress intensity of non-interpersonal situations (*M* = 7.42, *SD* = 1.25) was greater than that of interpersonal situations (*M* = 6.64, *SD* = 1.46) among European Canadians, *t*(52) = 2.97, *p* = .005, *d* = 0.408, 95% CI = [0.126, 0.687], whereas the perceived stress intensity of interpersonal (*M* = 6.96, *SD* = 1.37) and non-interpersonal situations (*M* = 7.04, *SD* = 1.54) did not differ among Japanese participants, *t*(49) = 0.33, *p* = .744, *d* = 0.047, 95% CI = [−0.231, 0.324], suggesting that Japanese participants perceived interpersonal situations as equally stressful as non-interpersonal situations (Figure 1).

#### Cultural differences in perceived frequency of situations

**Figure 2.**
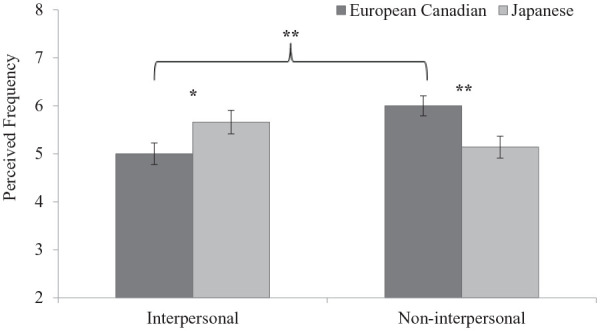
Cultural differences in perceived frequency of situations in Study 1. *Note.* Error bars depict standard errors. *p < .05. **p < .01 (two-tailed).

A 2 (Culture: European Canadian vs. Japanese; between-Ss) × 2 (Situation: interpersonal vs. non-interpersonal; within-Ss) mixed factorial ANOVA yielded nonsignificant main effects of situation, *F*(1, 101) = 1.31, *p* = .255, $\eta_{p}^{2}$ = .013, and culture, *F* < 1, *p* = .681, $\eta_{p}^{2}$ = .002. There was a significant Culture × Situation interaction, *F*(1, 101) = 13.15, *p* < .001, $\eta_{p}^{2}$ = .115 [.035, .215]. Simple-effects analyses revealed that European Canadians (*M* = 6.00, *SD* = 1.52) perceived non-interpersonal situations as more frequent than Japanese participants (*M* = 5.14, *SD* = 1.62), *t*(101) = 2.68, *p* = .008, *d* = 0.529, 95% CI = [0.135, 0.921], whereas Japanese participants (*M* = 5.66, *SD* = 1.73) perceived interpersonal situations as more frequent than European Canadians (*M* = 5.00, *SD* = 1.63), *t*(101) = 2.06, *p* = .042, *d* = 0.406, 95% CI = [0.015, 0.796]. Similar to the patterns shown in perceived intensity of situations, paired *t* tests for each culture revealed that European Canadians evaluated non-interpersonal situations (*M* = 6.00, *SD* = 1.52) as more frequent than interpersonal ones (*M* = 5.00, *SD* = 1.63), *t*(52) = 3.39, *p* = .001, *d* = 0.466, 95% CI = [0.180, 0.747], whereas Japanese participants perceived interpersonal situations (*M* = 5.66, *SD* = 1.73) and non-interpersonal situations (*M* = 5.14, *SD* = 1.62) as equally frequent, *t*(49) = 1.75, *p* = .086, *d* = 0.248, 95% CI = [−0.035, 0.528] (Figure 2).

#### Cultural variations in psychological versus physical word use

We employed Pennebaker’s Linguistic Inquiry and Word Count (Pennebaker et al., 2015) to quantify the frequency of *psychological words* that referred to emotional states (e.g., worry) and *physical words* that were classified under sensory and perceptual processes (e.g., heard) and physical states and functions (e.g., tiresome). All word counts are shown as a percentage of the total number of words, controlling for the length of the writing sample.

**Figure 3.**
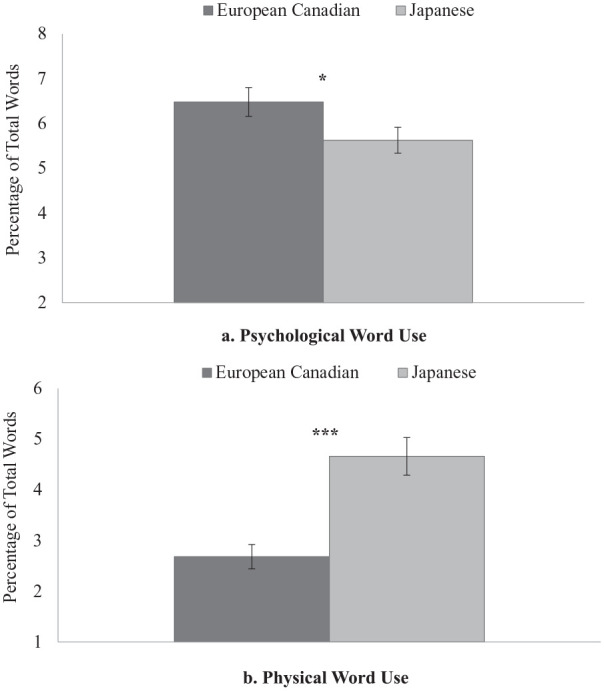
Cultural variations in psychological versus physical word use in Study 1. *Note.* Error bars depict standard errors. *p < .05. ***p < .001(two-tailed).

We compared mean-level differences of the counts of psychological and physical words between European Canadian and Japanese samples using *t* tests (Figure 3). Results showed that European Canadians (*M* = 6.48, *SD* = 2.86) used psychological words more frequently in describing their stress experiences compared with Japanese participants (*M* = 5.63, *SD* = 2.58), *t*(158) = 1.98, *p* = .049, *d* = 0.313, 95% CI = [0.001, 0.624]. In contrast, Japanese participants (*M* = 4.66, *SD* = 3.32) used physical words more often than European Canadians (*M* = 2.68, *SD* = 2.15), *t*(158) = 4.47, *p* < .001, *d* = 0.707, 95% CI = [0.386, 1.025].

Overall, the findings from Study 1 partially supported our predictions, showing that although perceived stress intensity of each situation was similar across cultures, perceived frequency of each situation differed between cultural groups. European Canadian undergraduates perceived non-interpersonal situations as occurring more frequently than Japanese undergraduates, whereas Japanese undergraduates evaluated interpersonal situations as more frequent than European Canadian undergraduates. Such situational perceptions persisted only among European Canadians: European Canadian undergraduates perceived higher stress intensity and frequency for non-interpersonal situations than interpersonal ones. However, Japanese undergraduates perceived a relatively equal intensity and frequency of each type. This situational perception among Japanese undergraduates shows that they pay roughly equal amounts of attention to the individual self and the social context, a common pattern among East Asians (Masuda et al., 2019). Our prediction for cultural variation in the focus of symptoms was also supported, revealing more frequent use of psychological words among European Canadian undergraduates versus physical words among Japanese undergraduates in their descriptions of stressful situations.

## Study 2

Study 1 demonstrated cultural differences in the perception of interpersonal and non-interpersonal situations when people assessed their daily stress experiences. However, it is unclear whether these situational perceptions would influence people’s perceived stress when they judge others’ experiences. Using the situations collected from Study 1, Study 2 was designed to measure how European Canadian and Japanese undergraduates would perceive different levels of stress in each type of situation. We examined whether we could observe similar patterns of situational perception in the reports of stress symptoms as obtained in Study 1: European Canadians would report more stress in non-interpersonal situations compared with interpersonal ones, whereas Japanese undergraduates would report as much stress in interpersonal situations as non-interpersonal ones, if not more. Comparing the two groups, we also expected to find greater stress from interpersonal situations among Japanese undergraduates and from non-interpersonal situations among European Canadians. In addition, we explored the question of whether a greater focus on psychological symptoms among European Canadians versus physical symptoms among Japanese undergraduates would appear, as shown in Study 1.

### Method

#### Participants

A priori power analysis using G*Power indicated that a sample size of 174 participants is needed to detect a three-way interaction with an effect size of $\eta_{p}^{2}$ = .06 and a power of .80 (Faul et al., 2009). We recruited 175 participants from two cultures: 88 European Canadian undergraduates (79.5% female; *M*_age_ = 18.97, *SD* = 1.47, range = 17–25) at the University of Alberta in Canada and 87 Japanese undergraduates (63.2% female; *M*_age_ = 19.54, *SD* = 1.16, range = 18–22) at Kobe University in Japan.

#### Measures and procedure

Of a total of 206 interpersonal and non-interpersonal situations generated in Study 1, we selected 160 situations that received ratings of stress intensity equal to or greater than “5 = *moderately stressful*” on a 9-point scale (*M* = 7.29, *SD* = 1.19; range = 5–9). We excluded about 20 situations from each cultural group that received low ratings of stress intensity (less than 5 on a 9-point scale) or that contained descriptions of multiple episodes. There were 40 from each of the four situation types derived from each cell of the 2 (Cultural Context) × 2 (Situation) design of Study 1.^3^ The 80 Canadian situations and the 80 Japanese situations were translated and back-translated by a team of bilingual Japanese–English researchers.

Furthermore, we modified culturally specific terms used in situations into general terms that could be applied for both cultures (e.g., replacing *Edmonton* with *a city*). We deleted any phrases that implied a resolution of situations (e.g., *Once the situation is over then you feel relief and the pressure is off*)^4^. We confirmed that each situation contained only one episode. We modified information from situations so that the main protagonist in each episode could be of either gender (e.g., *he or she*). Other than these changes, the original situational descriptions were kept intact.

We presented these situations to participants in a questionnaire format. To reduce the burden on the participants, we separated the sample of 160 situations into two versions of the questionnaire, in which each version contained 80 situations.^5^ In each questionnaire, the 80 situations were presented in a randomized order to participants. Each situation was followed by two questions: one about the likelihood of experiencing psychological symptoms of stress and the other about the likelihood of experiencing physical symptoms of stress, both on a scale from 1 (*not at all*) to 9 (*very much*). Internal consistencies for the measures of psychological symptoms (European Canadian: α = .97; Japanese: α = .95) and physical symptoms (European Canadian: α = .98; Japanese: α = .97) were excellent in both groups. Overall stress is a composite of psychological and physical symptoms, with high scores signifying higher levels of stress. Participants were asked to rate the two questions based on the definitions of psychological and physical symptoms as provided below and provide demographic information, such as gender and age5.

##### Psychological symptoms

When people are stressed in a given situation, they may focus on mental states that include feelings. For example, sometimes people feel hopeless, depressed, disappointed, or bitter. People may also feel afraid, sad, hostile, or aggressive. To avoid such distress, people may feel mentally numb or not feel anything. Furthermore, people may suffer from excessive worrying, including worrying about what others think of them.

##### Physical symptoms

When people are stressed in a given situation, they may focus on physical states. For example, sometimes people experience physical tension and stiffness in their body or shoulders. People may experience physiological responses such as dizziness, heart pounding, or sweating. People may also experience physical fatigue such as feeling weary and sleepy, or aches and pains in their stomach. Furthermore, people may suffer from sleeplessness, loss of appetite, itchiness, rashes, or a decrease in their ability to think as if they are “hazy” or “foggy.”

### Results

We conducted a 2 (Culture: European Canadian vs. Japanese; between-Ss) × 2 (Situation: interpersonal vs. non-interpersonal; within-Ss) × 2 (Symptom: psychological vs. physical; within-Ss) mixed factorial ANOVA on participants’ perceived likelihood of experiencing each symptom of stress. There were significant main effects of situation, *F*(1, 173) = 72.41, *p* < .001, $\eta_{p}^{2}$ = .295 [.204, .378], with people expressing greater overall stress in non-interpersonal situations (*M* = 5.50, *SD* = 1.10) than in interpersonal ones (*M* = 5.11, *SD* = 1.15), and symptom, *F*(1, 173) = 606.80, *p* < .001, $\eta_{p}^{2}$ = .778 [.732, .810], with people reporting greater psychological symptoms (*M* = 6.26, *SD* = 1.01) compared with physical symptoms (*M* = 4.36, *SD* = 1.33). However, the main effect of culture was nonsignificant, *F*(1, 173) = 2.82, *p* = .095, $\eta_{p}^{2}$ = .016 [.000, .060].

There was a significant Culture × Situation interaction, *F*(1, 173) = 99.54, *p* < .001, $\eta_{p}^{2}$ = .365 [.273, .444]. Simple-effects analyses showed that in interpersonal situations, Japanese participants (*M* = 5.48, *SD* = 0.91) perceived more overall stress compared with European Canadians (*M* = 4.75, *SD* = 1.26), *t*(173) = 3.07, *p* = .002, *d* = 0.464, 95% CI = [0.182, 0.761]. In non-interpersonal situations, European Canadians (*M* = 5.59, *SD* = 1.24) and Japanese participants (*M* = 5.41, *SD* = 0.94) perceived overall stress to a similar extent, *t*(173) = 0.78, *p* = .435, *d* = 0.118, 95% CI = [−0.179, 0.415]. These patterns of situational perception between cultures were consistently observed in psychological and physical symptoms (see Supplementary Material for the results of an additional analysis of the Culture × Situation interaction for each type of symptom). There was also a significant Culture × Symptoms interaction, *F*(1, 173) = 7.48, *p* = .007, $\eta_{p}^{2}$ = .041 [.007, .099]. Simple-effects analysis indicated that Japanese participants (*M* = 6.50, *SD* = 0.84) reported more psychological symptoms than European Canadians (*M* = 6.02, *SD* = 1.12), *t*(173) = 4.30, *p* < .001, *d* = 0.650, 95% CI = [0.353, 0.951], whereas there were no cultural differences in physical symptoms (European Canadians: *M* = 4.33, *SD* = 1.44 vs. Japanese: *M* = 4.38, *SD* = 1.22), *t*(173) = 0.08, *p* = .937, *d* = 0.012, 95% CI = [0.000, 0.079].

The Culture × Situation × Symptom interaction was significant, *F*(1, 173) = 13.82, *p* < .001, $\eta_{p}^{2}$ = .074 [.023, .142]. To break down the three-way interaction, we performed the analysis separately for the Situation × Symptom interaction in each cultural group: Among European Canadians, there were significant main effects of situation, *F*(1, 87) = 130.07, *p* < .001, $\eta_{p}^{2}$ = .599 [.488, .674], and symptom, *F*(1, 87) = 281.06, *p* < .001, $\eta_{p}^{2}$ = .764 [.690, .809]. Among Japanese participants, the main effect of situation was nonsignificant, *F*(1, 86) = 1.59, *p* = .211, $\eta_{p}^{2}$ = .018 [.000, .088], but we found a significant main effect of symptom, *F*(1, 86) = 325.61, *p* < .001, $\eta_{p}^{2}$ = .791 [.725, .831]. The Situation × Symptom interaction was significant for both European Canadians, *F*(1, 87) = 12.65, *p* = .001, $\eta_{p}^{2}$ = .127 [.037, .236], and Japanese participants, *F*(1, 86) = 73.29, *p* < .001, $\eta_{p}^{2}$ = .460 [.330, .556].

**Figure 4.**
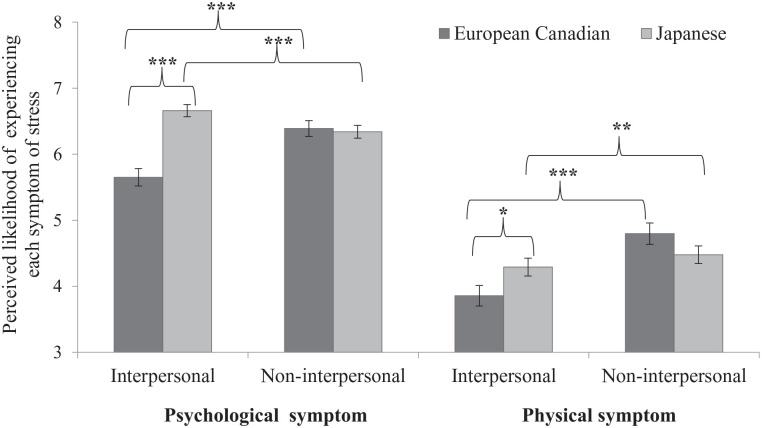
Means of psychological and physical symptoms depending on the interpersonal and the non-interpersonal situations among European Canadian and Japanese participants in Study 2. *Note.* Error bars depict standard errors. *p < .05. **p < .01. ***p < .001 (two-tailed).

A within-group analysis showed that relative to interpersonal situations (*M* = 5.65, *SD* = 1.24), non-interpersonal situations (*M* = 6.39, *SD* = 1.12) elicited more psychological symptoms among European Canadians, *t*(87) = 9.23, *p* < .001, *d* = 0.984, 95% CI = [0.727, 1.236]. Among Japanese participants, interpersonal situations (*M* = 6.66, *SD* = 0.86) elicited more psychological symptoms than non-interpersonal situations (*M* = 6.34, *SD* = 0.90), *t*(86) = 5.46, *p* < .001, *d* = 0.585, 95% CI = [0.356, 0.811] (left panel of Figure 4). In both groups, the effect of non-interpersonal situations (European Canadians: *M* = 4.80, *SD* = 1.52; Japanese: *M* = 4.48, *SD* = 1.25) was larger to elicit physical symptoms than that of interpersonal situations (European Canadians: *M* = 3.86, *SD* = 1.46; Japanese: *M* = 4.29, *SD* = 1.26), European Canadians: *t*(87) = 12.10, *p* < .001, *d* = 1.290, 95% CI = [1.005, 1.572]; Japanese: *t*(86) = 2.98, *p* = .004, *d* = 0.320, 95% CI = [0.103, 0.534] (right panel of Figure 4).

Overall, Study 2 replicated the patterns of situational perceptions shown in Study 1 and extended them to reveal the relationship between culture, situation, and symptom. Specifically, we found the expected patterns of situational perception for psychological symptoms within each culture. European Canadian undergraduates perceived more psychological symptoms in non-interpersonal situations compared with interpersonal situations, whereas Japanese undergraduates perceived more psychological symptoms in interpersonal situations than in non-interpersonal situations. However, both groups perceived more physical symptoms in non-interpersonal situations compared with interpersonal situations. We also confirmed that Japanese undergraduates perceived more psychological and physical symptoms of stress than European Canadians did in interpersonal situations, whereas there was no cultural difference in perceived stress in non-interpersonal situations. Contrary to the text analysis results in Study 1, however, Japanese undergraduates reported more psychological symptoms when evaluating others’ experiences compared with European Canadians, but were similar in their reports of physical symptoms.

## Study 3

Study 3 aimed to replicate the results of Study 2 for the validity of the findings, using a much-simplified definition of stress symptoms, which allowed participants to report their symptoms spontaneously, and assess whether cultural differences in perceived stress from interpersonal situations could be explained by social orientations. We attempted to replicate Study 2 with satisfaction scores. We expected that compared with European Canadians, Japanese undergraduates would report more psychological and/or physical symptoms from interpersonal situations, mediated by their weaker independence and/or stronger interdependence. In addition, we explored whether these associations would predict lower levels of life satisfaction (see Supplementary Material for the results on the relationships among culture, independence, interpersonal stress, and life satisfaction).

### Method

#### Participants

A priori Monte Carlo power analysis (Schoemann et al., 2017) informed that a sample size of 220 participants is required to detect indirect effects with two parallel mediators with 80% power. A sensitivity power analysis with G*Power showed that with an alpha of 5% and 80% power, the sample size was sufficient to detect an effect size of *f* = 0.22 for the three-way interaction and *f* = 0.19 for either of the two-way interactions. We recruited 223 participants from two cultures: 113 European Canadian undergraduates (63.7% female; *M*_age_ = 19.35, *SD* = 2.23, range = 17–29) at the University of Alberta in Canada and 110 Japanese undergraduates (55.5% female; *M*_age_ = 19.86, *SD* = 1.53, range = 18–23) at Kobe University in Japan.

#### Measures and procedure

Compared with the instructions in Study 2, we simplified our instructions so that participants spontaneously responded to the provided 80 (40 Canadian and 40 Japanese) situations from Version 1 of Study 2.^6^ In each situation, participants were asked to rate two questions, “When you imagine yourself in the situation, how likely would you be *mentally stressed out*? and how likely would you be *physically tired*?” on a scale from 1 (*not at all*) to 9 (*very much*). We counterbalanced the presentation of these questions to rule out order effects.^7^ Internal consistencies for the measures of psychological symptoms (European Canadian: α = .98; Japanese: α = .97) and physical symptoms (European Canadian: α = .98; Japanese: α = .98) were excellent in both cultures. Overall stress is a composite of psychological and physical symptoms, with high scores signifying higher levels of stress.

#### Social orientations

The 23-item *Self-Construal Scale* (Kim et al., 2003) was used to examine participants’ independent and interdependent scores on a scale from 1 (*strongly disagree*) to 7 (*strongly agree*). The independence measure consists of 13 items (e.g., “It is important for me to act as an independent person”) and the interdependence measure includes 10 items (e.g., “I am careful to maintain harmony in my group”). We dropped one item “Having a lively imagination is important to me” from the independence measure and two items “It is important to consult close friends and get their ideas before making decisions” and “If my brother or sister fails, I feel responsible” from the interdependence measure, due to poor loadings on internal consistency analyses (corrected item-total *r*s < .30). Internal consistencies for the measures of independence (European Canadian: α = .79; Japanese: α = .79) and interdependence (European Canadian: α = .73; Japanese: α = .81) were adequate across cultures. Following theoretical predictions that independence and interdependence are orthogonal dimensions (Singelis, 1994), we created the mean scores of independence and interdependence subscale separately, with higher scores indicating stronger independent or interdependent orientation. Supporting this notion, we found nonsignificant correlations between these constructs for both groups (European Canadian: *r* = −.07, *p* = .497; Japanese: *r* = .11, *p* = .267). Participants also provided demographic information, including gender and age.^7^

### Results

A 2 (Culture: European Canadian vs. Japanese; between-Ss) × 2 (Situation: interpersonal vs. non-interpersonal; within-Ss) × 2 (Symptom: psychological vs. physical; within-Ss) mixed factorial ANOVA was conducted on participants’ perceived likelihood of experiencing each symptom of stress. There were significant main effects of culture, *F*(1, 221) = 10.84, *p* = .001, c = .047 [.012, .099], with more overall stress among Japanese participants (*M* = 5.68, *SD* = 1.06) than European Canadians (*M* = 5.19, *SD* = 1.14); situation, *F*(1, 221) = 203.11, *p* < .001, $\eta_{p}^{2}$ = .479 [.402, .541], with more overall stress from non-interpersonal (*M* = 5.73, *SD* = 1.16) than interpersonal situations (*M* = 5.14, *SD* = 1.22); and symptom, *F*(1, 221) = 567.53, *p* < .001, $\eta_{p}^{2}$ = .720 [.671, .756], with more psychological symptoms (*M* = 6.32, *SD* = 1.10) than physical symptoms (*M* = 4.54, *SD* = 1.40).

Although there was no significant Culture × Symptom interaction, *F*(1, 221) = 0.10, *p* = .754, $\eta_{p}^{2}$ = .000 [.000, .019], there was a significant Culture × Situation interaction, *F*(1, 221) = 119.94, *p* < .001, c = .352 [.270, .423]. Simple-effects analyses showed that in interpersonal situations, Japanese participants (*M* = 5.61, *SD* = 1.07) perceived greater overall stress compared with European Canadians (*M* = 4.68, *SD* = 1.19), *t*(221) = 6.08, *p* < .001, *d* = 0.815, 95% CI = [0.541, 1.087]. In non-interpersonal situations, European Canadians (*M* = 5.70, *SD* = 1.19) and Japanese participants (*M* = 5.75, *SD* = 1.13) perceived overall stress to a similar extent, *t*(221) = 0.27, *p* = .789, *d* = 0.036, 95% CI = [−0.227, 0.299]. These patterns of situational perception between cultures were consistently observed in psychological and physical symptoms (see Supplementary Material for the results of an additional analysis of the Culture × Situation interaction for each type of symptom).

There was a significant Culture × Situation × Symptom interaction, *F*(1, 221) = 18.04, *p* < .001, $\eta_{p}^{2}$ = .076 [.022, .149]. We then performed the analysis separately for the Situation × Symptom interaction in each culture. There was a significant main effect of situation for European Canadians, *F*(1, 112) = 272.40, *p* < .001, $\eta_{p}^{2}$ = .709 [.618, .768], and Japanese participants, *F*(1, 109) = 6.61, *p* = .012, $\eta_{p}^{2}$ = .057 [.003, .157]. There was also a significant main effect of symptom for European Canadians, *F*(1, 112) = 271.51, *p* < .001, $\eta_{p}^{2}$ = .708 [.617, .767], and Japanese participants, *F*(1, 109) = 297.09, *p* < .001, $\eta_{p}^{2}$ = .732 [.645, .787]. The Situation × Symptom interaction was significant for both European Canadians, *F*(1, 112) = 58.483, *p* < .001, $\eta_{p}^{2}$ = .343 [.206, .459], and Japanese participants, *F*(1, 109) = 223.593, *p* < .001, $\eta_{p}^{2}$ = .672 [.571, .739].

**Figure 5.**
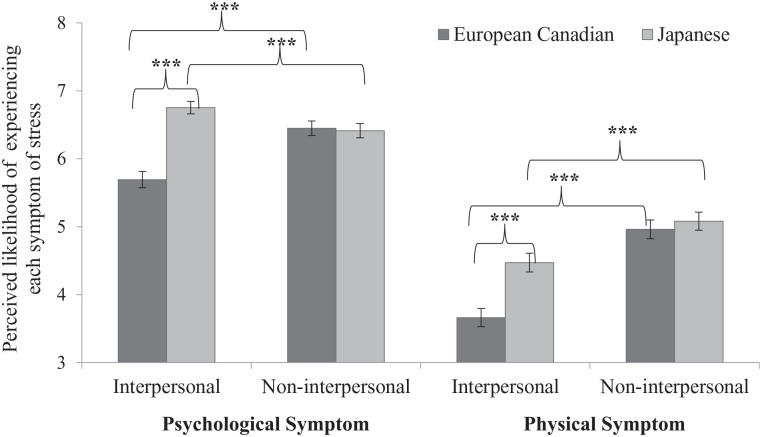
Means of psychological and physical symptoms depending on the interpersonal and the non-interpersonal situations among European Canadian and Japanese participants in Study 3. *Note.* Error bars depict standard errors. ***p < .001 (two-tailed).

A within-group analysis showed that relative to interpersonal situations (*M* = 5.69, *SD* = 1.28), non-interpersonal situations (*M* = 6.45, *SD* = 1.14) elicited more psychological symptoms among European Canadians, *t*(112) = 12.03, *p* < .001, *d* = 1.131, 95% CI = [0.893, 1.366]. In contrast, among Japanese participants, interpersonal situations (*M* = 6.75, *SD* = 0.97) elicited more psychological symptoms than non-interpersonal situations (*M* = 6.41, *SD* = 1.09), *t*(109) = 5.28, *p* < .001, *d* = 0.503, 95% CI = [0.304, 0.700] (left panel of Figure 5). In both groups, the effect of non-interpersonal situations (European Canadians: M = 3.66, SD = 1.42; Japanese: M = 4.47, SD = 1.44), European Canadians: t(112) = 16.34, …, Japanese: t(109) = 10.52, …95% CI = [0.772, 1.231] (right panel of Figure 5).

#### Social orientations as mediators

Before conducting mediational analysis, we tested the measurement equivalence of the social orientation variable using multigroup confirmatory factor analysis, with the fully constrained model, in which all factor loadings and the intercepts of the indicators were set to be equal between the two groups. This process allows for a comparison of the relationship between constructs among different groups (Van de Vijver & Leung, 1997; see Supplemental Table S5 for each model’s fit information).

We then tested whether the association between culture (European Canadians = 0, Japanese = 1) and psychological or physical symptoms of stress elicited from interpersonal situations was mediated by independence and interdependence. Here, we did not test for a mediation effect in symptoms from non-interpersonal situations as cultural differences only appeared in interpersonal situations. To this end, two multiple mediation analyses (PROCESS Model 4) were conducted with 5,000 bootstrapping procedures (Preacher & Hayes, 2008). We tested four indirect effects^8^ and found one significant indirect effect.

**Figure 6.**
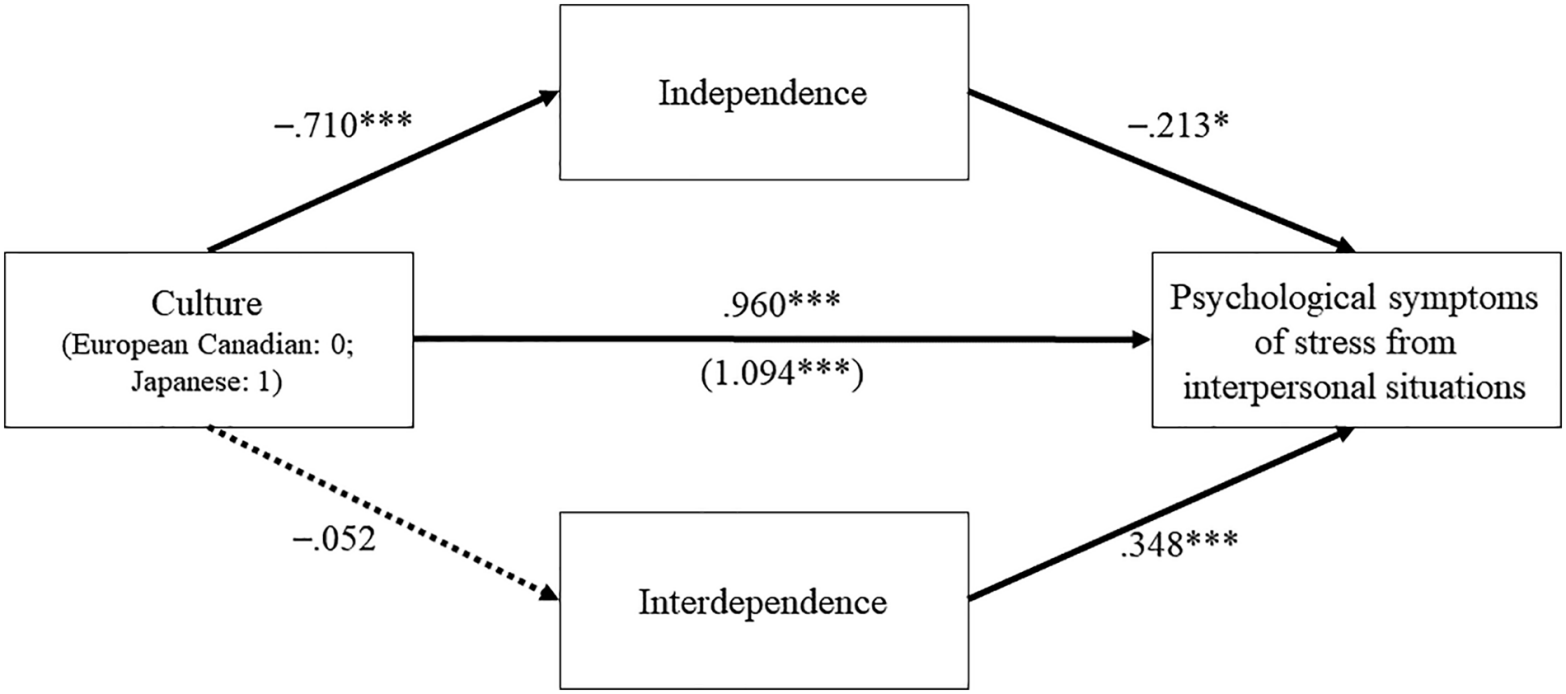
Multiple mediation analyses, Study 3. *Note.* Unstandardized regression coefficients. Indirect effect via independence = .151, 95% CI = [.013, .308]; indirect effect via interdependence = −.018, 95% CI = [−.105, .058]. The number inside the brackets is the total effect (c) and the number outside the brackets is the direct effect (c′). *p < .05. ***p < .001 (two-tailed).

The results based on the unstandardized regression coefficients showed lower independence scores among Japanese participants than European Canadians, *b* = −.710, *p* < .001, 95% CI = [−.907, −.514]. However, there was no cultural difference in the levels of interdependence, *b* = −.052, *p* = .644, 95% CI = [−.274, 170], which explains a nonsignificant indirect effect of culture on psychological symptoms from interpersonal situations via interdependence: the indirect effect = −.018, 95% CI = [−.105, .058]. As predicted, lower independence and higher interdependence were associated with more psychological symptoms from interpersonal situations, independence: *b* = −.213, *p* = .032, 95% CI = [−.408, −.019]; interdependence: *b* = .348, *p* < .001, 95% CI = [.175, .520]. More importantly, we found a significant indirect effect of culture on psychological symptoms from interpersonal situations via independence, the indirect effect = .151, 95% CI = [.013, .308] (Figure 6). This significant indirect effect suggests that cultural differences in psychological symptoms of stress arising from interpersonal situations are partially explained by individuals’ independence scores, which indicated that Japanese undergraduates reported greater psychological symptoms in interpersonal situations than European Canadians, due to their weaker orientation toward independence. In addition, the results of our exploratory analyses indicated that these associations predicted lower levels of life satisfaction (see Supplemental Figure S1).

In sum, the findings from Study 3 are largely consistent with those of Study 2. We provided evidence for the different extent to which the situational context moderates perceived symptoms of stress within each culture: European Canadians perceived more psychological symptoms arising from non-interpersonal situations compared with interpersonal situations, whereas Japanese undergraduates perceived more psychological symptoms from interpersonal situations than non-interpersonal situations. Regardless of culture, people perceived more physical symptoms in non-interpersonal situations than in interpersonal situations. In addition, Japanese undergraduates reported more psychological and physical symptoms than European Canadians did in interpersonal situations, with no cultural difference in perceived symptoms in non-interpersonal situations. Moreover, we demonstrated weaker independent orientations partially mediated the association between being Japanese undergraduates (vs. European Canadians) and greater psychological symptoms from interpersonal situations.

## General Discussion

Using a situation sampling method, the current paper advances our understanding of the relationship between culture, daily situational contexts, and stress symptoms. Our findings showed that depending on the type of stressful situation (interpersonal vs. non-interpersonal), European Canadians and Japanese undergraduates perceived the intensity and frequency of each type of situation differently. Such situational perceptions were also manifested in how much undergraduates report psychological and physical symptoms of stress under the situations.

In Study 1, within-culture comparisons partially supported our prediction that European Canadians perceive non-interpersonal situations as more stressful and frequently occurring compared with interpersonal ones. However, contrary to our prediction, Japanese undergraduates perceived similar stress intensity and frequency for both situations, which may be due to their equal prioritization of personal and interpersonal goals. Between-culture comparisons revealed the expected cultural differences only in perceived frequency: European Canadians perceived stressful non-interpersonal situations as more frequent compared with Japanese undergraduates, whereas Japanese undergraduates perceived stressful interpersonal situations as more frequent than European Canadians. However, there was no cultural difference in perceived stress intensity for each type of situation, demonstrating that participants are more inclined to report the daily situational context that they perceived as “moderately” or “very stressful” when asked to think of each one. In addition, the results from the text analysis supported our prediction of a greater focus on psychological symptoms among European Canadians and a greater focus on physical symptoms among Japanese undergraduates.

In Studies 2 and 3, the expected patterns of situational perceptions in each culture were shown only in the reports of psychological symptoms among the participants: European Canadians perceived more psychological symptoms when they imagined themselves experiencing stressful non-interpersonal situations over interpersonal ones, whereas Japanese undergraduates perceived more psychological symptoms when they imagined themselves in interpersonal situations rather than non-interpersonal ones. On the contrary, in the reports of physical symptoms, both cultural groups perceived more physical symptoms in non-interpersonal situations than in interpersonal situations, which is partially consistent with our predictions. Indeed, European Canadians continually perceived more physical symptoms in non-interpersonal situations than in interpersonal situations, but unexpectedly. Japanese undergraduates showed greater physical symptoms in non-interpersonal situations than in interpersonal situations.

Between-culture comparisons showed that as predicted, Japanese undergraduates reported a greater likelihood of experiencing psychological and physical symptoms of stress than European Canadians did in interpersonal situations, but unexpectedly, there was no cultural difference in the perceived likelihood of experiencing either type of symptoms in non-interpersonal situations. Overall, the likelihood of experiencing psychological symptoms was rated higher than physical symptoms for both Japanese and European Canadian undergraduates. These results partially support our predictions and replicate those of prior studies that have shown greater psychological distress in interpersonal relationships among East Asians than North Americans (Hashimoto et al., 2012; Kirmayer, 1993),

In Study 3, we originally planned to examine cultural differences in perceived symptoms of stress arising from interpersonal versus non-interpersonal situations and hypothesized that these differences can be mediated by independent and/or interdependent orientation. Given no cultural differences in perceived symptoms from non-interpersonal situations, we focused instead on the indirect effects of independence and interdependence in the relationship between culture and symptoms from interpersonal situations. The results of our modified analysis plan partially supported our predictions, revealing the indirect effect of independence in the link between culture and psychological symptoms from interpersonal situations. We interpret these findings to show that weaker independence among Japanese undergraduates (vs. European Canadians) could explain why they perceived greater psychological symptoms from interpersonal situations. However, because of the absence of any association between culture and interdependence, we did not find the expected indirect effect of interdependence in the link between culture and psychological symptoms from interpersonal situations.

Theoretical patterns in social orientations proposed by Varnum et al. (2010) have shown discrepancy in our data. Our data showed differences between European Canadians and Japanese undergraduates in independence, with no differences in interdependence; thus, we found a significant mediation through independence rather than interdependence. We speculate on two conceptual and methodological limitations that did not allow us to fully capture more nuanced cultural differences in interdependence. First, culturally distinctive patterns in social orientations may not appear in between-country comparisons because interpretations of each item of the Self-Construal Scale differ by cultural nuances in language. In addition, social orientation is possibly multidimensional rather than two dichotomous dimensions (Vignoles et al., 2016). For example, Vignoles et al. proposed a 7-dimensional model of independence versus interdependence that consists of self-reliance versus dependence on others, self-containment versus connection to others, a desire to be different versus similar to others, self-interest versus commitment to others, consistency versus variability across situations, self-direction versus receptiveness to influence by others, and self-expression versus maintaining harmony with others. This 7-dimensional model may address more nuanced cultural differences in social orientations than the existing two-dimensional model.

Overall, most of the results are consistent across the three studies, as well as with our hypotheses. However, there were some unexpected patterns. For example, all studies showed greater overall stress from non-interpersonal situations compared with interpersonal situations across cultures. This relative priority given to non-interpersonal situations is due to the characteristics of the undergraduate samples, which is consistent with prior research, suggested that academic stress is often considered as chronic stress among undergraduates (Ribeiro et al., 2018). Also Studies 2 and 3 revealed significantly higher scores of psychological symptoms than of physical symptoms across cultures. This can be attributable to the procedure of the situation sampling method. Different from Study 1, Studies 2 and 3 asked participants to read other people’s stressful situations and to imagine themselves in those situations. In hindsight, this procedure could have directed the participants’ attention to the psychological aspects rather than the physical aspects of the protagonists’ stress experiences and applied this attention pattern to the judgment of their own stress level. Indeed, it is difficult to imagine another’s physical symptoms of stress and easier to guess their psychological symptoms. So, to go beyond our speculation, future studies should clearly differentiate between people’s subjective stress experiences and people’s judgment of others’ stress experiences and articulate whether there is any cultural bias in them.

### Implications

Our findings shed light on cultural influences in people’s perception of stressful situations, which guides their coping behaviors. Although prior studies on stress and coping have documented cultural differences in how people cope with stress and how different ways of coping influence health and well-being (Chun et al., 2006), our study question, “What types of situations do people from different cultures perceive as stressful in the first place?” has received limited attention to date. The current research, therefore, underscores how the interaction between a person and their culturally salient, immediate situation can be key in explaining cultural differences in people’s experiences of daily stress.

The current research has potential implications for the development of culturally appropriate intervention programs for the well-being and health of culturally diverse undergraduate students. Prior health care research has addressed the issue of de-emphasizing cultural values of Asian immigrants in Western practices (Kuo & Gingrich, 2005). Evidence about cultural differences in perceptions of stress provided by this research could inform culturally sensitive stress management programs for undergraduate students who hold different social orientations. For example, interpersonal stress management programs (e.g., conflict management training) could be effective at improving the well-being and health of Asian-descent undergraduate students (Inman & Yeh, 2007). This possibility should be further investigated by conducting more culturally sensitive explorations in clinical outcomes.

### Limitations and Future Directions

While cultural influences need to be analyzed in a multilevel approach integrating various situational and individual factors (Miyamoto, 2013), we primarily relied on ethnicity as a proxy for culture in Studies 1 and 2. Nonetheless, this limitation is addressed in Study 3, where we looked at relationships among ethnicity, culturally salient contexts, and individual-level social orientations. Also, our samples consisted only of undergraduate students. This may limit the generalizability of our findings to populations of different ages, given the evidence of age differences in daily stress experiences (Scott et al., 2013).

In Study 2, we provided the definition of psychological and physical symptoms with comprehensive examples to participants, but in Study 3, we simply asked participants to report the likelihood of “being mentally stressed out” and “being physically tired.” The differences in how the concept of each type of symptoms was presented to participants may have influenced the findings in the two studies. Nonetheless, the examples provided in Study 2 were considered as common psychological and physical symptoms of stress. As well, from the situations collected from Study 1, European Canadian and Japanese undergraduates used the comparable frequency of the psychological term *stressed out* and the physical term *tiredness* to describe their stress experiences.

Our findings from Study 3 are based on self-reported cross-sectional data, which have limitations with respect to testing causal mediation (Pek & Hoyle, 2016). Also, we did not include the construct of coping, which plays a key role in the link between stress and well-being. Although Study 3 showed that cultural differences in interpersonal stress were partially mediated by levels of independent social orientation, these effects can be influenced by coping. To fully capture the role of culture in stress and coping, our follow-up study will investigate cultural differences in ways of coping with different types of daily stressful situations and the role of social orientations in these cultural differences (Han et al., in press).

While we focused on cultural differences in perceptions of negative (stressful) interpersonal contexts, it is possible that such cultural differences may also appear in perceptions of positive social contexts. For example, Uchida et al. (2008) demonstrated a greater effect of perceived emotional support on well-being and physical health among Japanese undergraduates compared with European Americans. In line with the prior findings, we speculate that East Asians who are highly sensitive to interpersonal relationships may show greater psychological and/or physical reactions to positive interpersonal contexts than North Americans. Further investigation is required to confirm this speculation.

In addition, while our exploratory analyses show a negative association between interpersonal stress and life satisfaction, a growing body of literature addresses the positive effects of stress on emotions and motivations. In certain stressful situations, positive and negative emotions co-occur and a certain degree of stress motivates people to perform better (Folkman & Moskowitz, 2000) and resolve interpersonal conflicts (Markus & Lin, 1999). Future research should extend our findings by including outcome measures that may reveal nuances in how people with different social orientations construe the meaning of stress and well-being differently.

## Conclusion

Using a situation sampling method, the present studies explore cultural differences in people’s daily stress experiences. The present research provides evidence for the possibility that people from different cultures perceive daily interpersonal and non-interpersonal stress distinctively. Moreover, the present research demonstrates the role of social orientations in explaining cultural differences in levels of interpersonal stress.

## Research Data

sj-csv-2-psp-10.1177_01461672211070360 – for Cultural Differences in the Perception of Daily Stress Between European Canadian and Japanese Undergraduate Students Click here for additional data file. sj-csv-2-psp-10.1177_01461672211070360 for Cultural Differences in the Perception of Daily Stress Between European Canadian and Japanese Undergraduate Students by Hajin Lee, Takahiko Masuda, Keiko Ishii, Yuto Yasuda and Yohsuke Ohtsubo in Personality and Social Psychology Bulletin This article is distributed under the terms of the Creative Commons Attribution 4.0 License ( https://creativecommons.org/licenses/by/4.0/) which permits any use, reproduction and distribution of the work without further permission provided the original work is attributed as specified on the SAGE and Open Access pages ( https://us.sagepub.com/en-us/nam/open-access-at-sage).

sj-csv-3-psp-10.1177_01461672211070360 – for Cultural Differences in the Perception of Daily Stress Between European Canadian and Japanese Undergraduate Students Click here for additional data file. sj-csv-3-psp-10.1177_01461672211070360 for Cultural Differences in the Perception of Daily Stress Between European Canadian and Japanese Undergraduate Students by Hajin Lee, Takahiko Masuda, Keiko Ishii, Yuto Yasuda and Yohsuke Ohtsubo in Personality and Social Psychology Bulletin This article is distributed under the terms of the Creative Commons Attribution 4.0 License ( https://creativecommons.org/licenses/by/4.0/) which permits any use, reproduction and distribution of the work without further permission provided the original work is attributed as specified on the SAGE and Open Access pages ( https://us.sagepub.com/en-us/nam/open-access-at-sage).

sj-csv-4-psp-10.1177_01461672211070360 – for Cultural Differences in the Perception of Daily Stress Between European Canadian and Japanese Undergraduate Students Click here for additional data file. sj-csv-4-psp-10.1177_01461672211070360 for Cultural Differences in the Perception of Daily Stress Between European Canadian and Japanese Undergraduate Students by Hajin Lee, Takahiko Masuda, Keiko Ishii, Yuto Yasuda and Yohsuke Ohtsubo in Personality and Social Psychology Bulletin This article is distributed under the terms of the Creative Commons Attribution 4.0 License ( https://creativecommons.org/licenses/by/4.0/) which permits any use, reproduction and distribution of the work without further permission provided the original work is attributed as specified on the SAGE and Open Access pages ( https://us.sagepub.com/en-us/nam/open-access-at-sage).

sj-csv-5-psp-10.1177_01461672211070360 – for Cultural Differences in the Perception of Daily Stress Between European Canadian and Japanese Undergraduate Students Click here for additional data file. sj-csv-5-psp-10.1177_01461672211070360 for Cultural Differences in the Perception of Daily Stress Between European Canadian and Japanese Undergraduate Students by Hajin Lee, Takahiko Masuda, Keiko Ishii, Yuto Yasuda and Yohsuke Ohtsubo in Personality and Social Psychology Bulletin This article is distributed under the terms of the Creative Commons Attribution 4.0 License ( https://creativecommons.org/licenses/by/4.0/) which permits any use, reproduction and distribution of the work without further permission provided the original work is attributed as specified on the SAGE and Open Access pages ( https://us.sagepub.com/en-us/nam/open-access-at-sage).

sj-docx-1-psp-10.1177_01461672211070360 – Supplemental material for Cultural Differences in the Perception of Daily Stress Between European Canadian and Japanese Undergraduate Students Click here for additional data file. Supplemental material, sj-docx-1-psp-10.1177_01461672211070360 for Cultural Differences in the Perception of Daily Stress Between European Canadian and Japanese Undergraduate Students by Hajin Lee, Takahiko Masuda, Keiko Ishii, Yuto Yasuda and Yohsuke Ohtsubo in Personality and Social Psychology Bulletin

## Notes

1. Females (*M* = 7.18, *SD* = 0.97) perceived their situations as more stressful than males (*M* = 6.74, *SD* = 1.18), *F*(1, 99) = 4.13, *p* = .045, $\eta_{p}^{2}$ = .040 [.001, .119], but no Gender × Culture interaction in intensity, *F*(1, 99) < 1, *p* = .904. There were no gender differences, *F*(1, 99) < 1, *p* = .769, and Gender × Culture interaction in frequency, *F*(1, 99) < 1, *p* = .793. There were no age differences in intensity, *F*(7, 93) = 1.13, *p* = .352, and frequency, *F*(7, 93) < 1, *p* = .600.

2. We employed 90% confidence interval (CI) to calculate the CIs for partial eta-squared because it indicates statistical significance at .05 (Steiger, 2004). We used a syntax file that was originally crafted by Michael Smithson and modified by Karl Wuensch, http://core.ecu.edu/psyc/wuenschk/SPSS/SPSS-Programs.htm.

3. The main effect of cultural context was significant, *F*(1, 173) = 255.69, *p* < .001, $\eta_{p}^{2}$ = .596 [.521, .653], with more stress for Canadian (*M* = 5.57, *SD* = 1.09) versus Japanese (*M* = 5.04, *SD* = 1.12) situations. The Culture × Situation × Cultural Context interaction was significant, *F*(1, 173) = 27.644, *p* < .001, $\eta_{p}^{2}$ = .138 [.067, .217]. Both groups perceived greater stress from Canadian (European Canadians: *M* = 5.11, *SD* = 1.29; Japanese: *M* = 5.64, *SD* = 0.92) versus Japanese (European Canadians: *M* = 4.39, *SD* = 1.30; Japanese: *M* = 5.31, *SD* = 0.99) interpersonal situations. European Canadians perceived greater stress from Canadian (*M* = 6.12, *SD* = 1.29) versus Japanese (*M* = 5.07, *SD* = 1.26) non-interpersonal situations, whereas Japanese participants perceived similar levels of stress from both Canadian (*M* = 5.42, *SD* = 1.06) and Japanese (*M* = 5.40, *SD* = 0.91) non-interpersonal situations.

4. Only European Canadians reported situations that have already been resolved (20.75% of Canadian interpersonal situations; 24.53% of Canadian non-interpersonal situations). A chi-square test was performed and the relationship between culture and this tendency was significant, χ^2^(1, *N* = 206) = 25.63, *p* < .001.

5. There were no effects of version, *F*(1, 171) = 1.944, *p* = .165, and Version × Culture interaction, *F*(1, 171) = 0.015, *p =* .902. Females (*M* = 5.40, *SD* = 1.04) perceived more stress compared with males (*M* = 5.07, *SD* = 1.10), *F*(1, 171) = 6.42, *p* = .012, $\eta_{p}^{2}$ = .036 [.004, .092], but no Gender × Culture interaction, *F*(1, 171) = 1.77, *p* = .186. There was no age differences, *F*(8, 161) = 0.926, *p* = .496, but a marginally significant Age × Culture interaction, *F*(4, 161) = 2.35, *p* = .056, $\eta_{p}^{2}$ = .055 [.000, .101].

6. The main effect of cultural context was significant, *F*(1, 221) = 138.33, *p* < .001, $\eta_{p}^{2}$ = .385 [.304, .455], with more symptoms for Canadian (*M* = 5.60, *SD* = 1.13) versus Japanese (*M* = 5.26, *SD* = 1.20) situations. The Culture × Situation × Cultural Context interaction was significant, *F*(1, 221) = 127.99, *p* < .001, $\eta_{p}^{2}$ = .367 [.285, .438]. European Canadians perceived greater overall stress from Canadian (*M* = 4.88, *SD* = 1.20) versus Japanese (*M* = 4.48, *SD* = 1.24) interpersonal situations, *t*(112) = 8.10, *p* < .001, *d* = 0.762, 95% CI = [0.551, 0.970]. Japanese participants perceived similar levels of stress from both Canadian (*M* = 5.64, *SD* = 1.11) and Japanese (*M* = 5.59, *SD* = 1.07) interpersonal situations, *t*(109) = 1.11, *p* = .270, *d* = 0.106, 95% CI = [−0.082, 0.293]. European Canadians perceived greater stress to Canadian (*M* = 6.25, *SD* = 1.27) versus Japanese (*M* = 5.16, *SD* = 1.18) non-interpersonal situations, *t*(112) = 19.23, *p* < .001, *d* = 1.809, 95% CI = [1.507, 2.107], whereas Japanese participants perceived greater stress to Japanese (*M* = 5.85, *SD* = 1.11) versus Canadian (*M* = 5.64, *SD* = 1.20) non-interpersonal situations, *t*(109) = 4.43, *p* < .001, *d* = 0.423, 95% CI = [0.227, 0.617].

7. There were no effects of order, *F*(1, 219) = 0.006, *p* = .936, and Order × Culture interaction, *F*(1, 219) = 0.280, *p* = .597. Females (*M* = 5.56, *SD* = 1.08) perceived greater stress compared with males (*M* = 5.24, *SD* = 1.18), *F*(1, 219) = 6.30, *p* = .013, $\eta_{p}^{2}$ = .028 [.003, .073], but Gender × Culture interaction was nonsignificant, *F*(1, 219) = 1.98, *p* = .161. There were no effects of age, *F*(11, 205) = 1.53, *p* = .125, and Culture × Age interaction, *F*(4, 205) = 1.57, *p* = .183.

8. Due to the absence of any association between social orientations and physical symptoms of stress from interpersonal situations (independence: *b* = −.020, *p* = .877, 95% CI = [−.280, .239]; interdependence: *b* = .135, *p* = .246, 95% CI = [−.094, .365]), the indirect effects of culture on physical symptoms via social orientations were nonsignificant (independence: indirect effect = .015, 95% CI = [−.186, .218]; interdependence: indirect effect = −.007, 95% CI = [−.061, .031]).
