# Supplementary material for: Cultural Differences in the Perception of Daily Stress Between European Canadian and Japanese Undergraduate Students
Source: Pers Soc Psychol Bull. 2022 Feb 25;49(4):571–84. doi: 10.1177/01461672211070360 (PMC9989219; doi:10.1177/01461672211070360)
Supplement: sj-docx-1-psp-10.1177_01461672211070360 – Supplemental material for Cultural Differences in the Perception of Daily Stress Between European Canadian and Japanese Undergraduate Students [file sj-docx-1-psp-10.1177_01461672211070360.docx]

**Supplemental Material**

**Supplementary Table 1.** Respondents’ High-ranked Sources of Stress across Cultures

| Sources of *interpersonal stress* of **European Canadian** undergraduates | Percentage (%) |
| --- | --- |
| Responsibility in school or work | 30% |
| Family problems | 17.5% |
| Disagreement with peers | 17.5% |
| Talk in front of class | 10% |
| Performance on interview | 7.5% |
| Forming new relationships | 7.5% |
| Living adjustments (such as roommate issues) | 5% |
| Conflicts with boss or co-workers | 5% |
| Sources of *non-interpersonal stress* of **European Canadian** undergraduates | Percentage (%) |
| Performance on exams | 65% |
| Concern for future | 7.5% |
| Lack of personal ability | 7.5% |
| Late for school | 5% |
| Technical issue (i.e., computer, car) | 5% |
| Health issue | 5% |
| Being lost | 2.5% |
| Workload in school | 2.5% |
| Sources of *interpersonal Stress* of **Japanese** undergraduates | Percentage (%) |
| Concern for a friend’s behavior | 25% |
| Sense of social isolation | 20% |
| Potential conflict with others in school or work | 20% |
| Social responsibility in school or work | 17.5% |
| Disagreement with friends | 10% |
| Family problems | 5% |
| Frustration from not meeting others’ expectations | 2.5% |
| Sources of *non-interpersonal stress* of **Japanese** undergraduates | Percentage (%) |
| Academic performance | 25% |
| Transportation problems | 17.5% |
| Lack of personal ability | 12.5% |
| Social responsibility | 7.5% |
| Health issue | 7.5% |
| Financial concern | 5% |
| Concern for future | 5% |
| Noise in neighborhood | 5% |
| Technical problems | 5% |
| Late for class | 5% |
| Disappointment from the situation | 2.5% |
| Workload in school | 2.5% |

**Supplementary Table 2.** *Means, Standard Deviations, and Correlations of Key Variables among European Canadian and Japanese undergraduates (Study 1).*

| Variable | 1 | 2 | 3 | 4 | 5 | 6 |
| --- | --- | --- | --- | --- | --- | --- |
| 1. Perceived intensity for interpersonal situations | — |  |  |  |  |  |
| 1. Perceived intensity for non-interpersonal situations | .148 | — |  |  |  |  |
| 1. Perceived frequency for interpersonal situations | .194^*^ | -.06 | — |  |  |  |
| 1. Perceived frequency for non-interpersonal situations | -.10 | .25^*^ | .09 | — |  |  |
| 1. Mental word use | -.16 | -.15 | .00 | -.00 | — |  |
| 1. Physical word use | -.01 | -.02 | .10 | -.12 | .06 | — |
| European Canadian (*n* = 53) |  |  |  |  |  |  |
| *M* | 6.64 | 7.42 | 5.00 | 6.00 | 6.48 | 2.68 |
| *SD* | 1.46 | 1.25 | 1.63 | 1.52 | 2.86 | 2.15 |
| Japanese (*n* = 50) |  |  |  |  |  |  |
| *M* | 6.96 | 7.04 | 5.66 | 5.14 | 5.63 | 4.66 |
| *SD* | 1.37 | 1.54 | 1.73 | 1.62 | 2.58 | 3.32 |

*Note.* *M* and *SD* are used to represent mean and standard deviation, respectively.

^*^*p* < .05 ^**^*p* < .01 ^***^*p* < .001 (two-tailed).

**Supplementary Table 3.** *Means, Standard Deviations, and Correlations of Key Variables among European Canadian and Japanese undergraduates (Study 2).*

| Variable | 1 | 2 | 3 | 4 |
| --- | --- | --- | --- | --- |
| 1. Psychological symptoms from interpersonal situations | — |  |  |  |
| 1. Physical symptoms from interpersonal situations | .63^***^ | — |  |  |
| 1. Psychological symptoms from non-interpersonal situations | .71^***^ | .59^***^ | — |  |
| 1. Physical symptoms from non-interpersonal situations | .40^***^ | .85^***^ | .66^***^ | — |
| European Canadian (*n* = 88) |  |  |  |  |
| *M* | 5.65 | 3.86 | 6.39 | 4.80 |
| *SD* | 1.24 | 1.46 | 1.12 | 1.52 |
| Japanese (*n* = 87) |  |  |  |  |
| *M* | 6.66 | 4.29 | 6.34 | 4.48 |
| *SD* | 0.86 | 1.26 | 0.90 | 1.25 |

*Note.* *M* and *SD* are used to represent mean and standard deviation, respectively.

^*^*p* < .05 ^**^*p* < .01 ^***^*p* < .001 (two-tailed).

**Additional Analysis in Study 2**

We ran the analysis for the Culture X Situation interaction in each symptom. In the ratings of psychological symptoms (left panel of Figure 4), there were significant main effects of Culture, F(1, 173) = 10.321, p = .002, ηp2 = .056 [.013, .120], and Situation, F(1, 173) = 17.77, p < .001, ηp2 = .093 [.035, .166]. These effects were qualified by a significant Culture X Situation interaction, F(1, 173) = 113.40, p < .001, ηp2 = .396 [.304, .473]. Simple-effects analysis revealed that in interpersonal situations, Japanese participants (M = 6.66, SD = 0.86) perceived greater psychological symptoms than European Canadians (M = 5.65, SD = 1.24), t(173) = 6.41, p < .001, d = .969, 95%CI = [.654, 1.281]. In non-interpersonal situations, European Canadians (M = 6.39, SD = 1.12) and Japanese participants (M = 6.34, SD = 0.90) perceived a similar level of psychological symptoms, t(173) = 0.31, p = .378, d = .047, 95%CI = [−.249, .344].

For physical symptoms (right panel of Figure 4), we found a significant main effect of Situation, F(1, 173) = 127.28, p < .001, ηp2 = .424 [.333, .499], but not Culture, F(1, 173) = .08, p = .777, ηp2 = .000 [.000, .017]. The Culture X Situation interaction was significant, F(1, 173) = 56.99, p < .001, ηp2 = .248 [.160, .331]. Simple-effects analysis indicated that in interpersonal situations, Japanese participants (M = 4.29, SD = 1.26) perceived greater physical symptoms than European Canadians (M = 3.86, SD = 1.46), t(173) = 2.09, p = .019, d = .316, 95%CI = [.017, .613], while in non-interpersonal situations, European Canadians (M = 4.80, SD = 1.52) and Japanese participants (M = 4.48, SD = 1.25) perceived a similar level of physical symptoms, t(173) = 1.54, p = .063, d = .232, 95%CI = [−.065, .529].

**Supplementary Table 4.** *Means, Standard Deviations, and Correlations of Key Variables among European Canadian and Japanese undergraduates (Study 3).*

| Variable | 1 | 2 | 3 | 4 | 5 | 6 | 7 |
| --- | --- | --- | --- | --- | --- | --- | --- |
| 1. Psychological symptoms from interpersonal situations | — |  |  |  |  |  |  |
| 1. Physical symptoms from interpersonal situations | .60^***^ | — |  |  |  |  |  |
| 1. Psychological symptoms from non-interpersonal situations | .74^***^ | .50^***^ | — |  |  |  |  |
| 1. Physical symptoms from non-interpersonal situations | .50^***^ | .85^***^ | .65^***^ | — |  |  |  |
| 1. Independent orientation | -.29^***^ | -.12 | -.12 | -.03 | — |  |  |
| 1. Interdependent orientation | .22^***^ | .10 | .24^***^ | .15^*^ | .06 | — |  |
| 1. Life satisfaction | -.38^***^ | -.20^**^ | -.20^**^ | -.12 | .37^***^ | .04 | — |
| European Canadian (*n* = 113) |  |  |  |  |  |  |  |
| *M* | 5.69 | 3.66 | 6.45 | 4.96 | 5.54 | 4.76 | 4.82 |
| *SD* | 1.28 | 1.42 | 1.14 | 1.47 | 0.67 | 0.79 | 1.16 |
| Japanese (*n* = 110) |  |  |  |  |  |  |  |
| *M* | 6.75 | 4.47 | 6.41 | 5.08 | 4.83 | 4.71 | 3.91 |
| *SD* | 0.97 | 1.44 | 1.09 | 1.39 | 0.80 | 0.88 | 1.17 |

*Note.* *M* and *SD* are used to represent mean and standard deviation, respectively. ^*^*p* < .05 ^**^*p* < .01 ^***^*p* < .001 (two-tailed).

**Additional Analysis in Study 3**

We ran the analyses for the Culture X Situation interaction in each symptom. For psychological symptoms (left panel of Figure 5), there was a significant main effect of Culture, *F*(1, 221) = 12.62, *p* < .001, η_p_^2^ = .054 [.016, .109], and Situation, *F*(1, 221) = 21.44, *p* < .001, η_p_^2^ = .088 [.037, .152], which were qualified by a significant Culture X Situation interaction, *F*(1, 221) = 148.32, *p* < .001, η_p_^2^ = .402 [.321, .470]. Simple-effects analysis revealed that in interpersonal situations, Japanese participants (*M* = 6.75, *SD* = 0.97) perceived more psychological symptoms than European Canadians (*M* = 5.69, *SD* = 1.28), *t*(221) =7.02, *p* < .001, *d* = .941, 95%CI = [.663, 1.217]. In non-interpersonal situations, European Canadian (*M* = 6.45, *SD* = 1.14) and Japanese participants (*M* = 6.41, *SD* = 1.09) perceived a similar level of psychological symptoms, *t*(221) = 0.24, *p* = .809, *d* = .032, 95%CI = [−.230, .295].

For physical symptoms (right panel of Figure 5), we found significant main effects of Culture, *F*(1, 221) = 6.27, *p* = .013, η_p_^2^ = .028 [.003, .072], and Situation, *F*(1, 221) = 373.13, *p* < .001, η_p_^2^ = .628 [.566, .675]. The Culture X Situation interaction was significant, *F*(1, 221) = 48.89, *p* < .001, η_p_^2^ = .181 [.110, .254]. Simple-effects analysis indicated more physical symptoms among Japanese participants (*M* = 4.47, *SD* = 1.44) than European Canadians (*M* = 3.66, *SD* = 1.42) in interpersonal situations, *t*(221) = 4.22, *p* < .001 , *d* = .565, 95%CI = [.297, .832], but both groups had a similar level of physical symptoms in non-interpersonal situations (*M*_EC_ = 4.96, *SD* = 1.47; *M*_J_ = 5.08, *SD* = 1.39), *t*(221) = −0.79, *p* = .431, *d* = .106, 95%CI = [−.157, .368].

**Test of Measurement Invariance**

We tested the measurement equivalence of model variables using multi-group confirmatory factor analysis (CFA), with the fully constrained model, in which all factor loadings and the intercepts of the indicators were set equal between the two cultural groups. This process allows a meaningful comparison of the relationship between psychological constructs among different cultural groups (Van de Vijver & Leung, 1997). We first constructed a configural invariance model where factor loadings and intercepts of the indicator variables were freely estimated. These initial models for all the variables for the European Canadian and Japanese groups suggested a good fit. Next, we constrained the factor loadings for each indicator for equality between cultural groups to compute a model with factorial invariance. A final test of measurement invariance was conducted by constraining the intercepts of each indicator to equality between the two groups. **Supplementary Table 5** contains fit information for each model. Each model variable under investigation in this study achieved metric invariance, signifying they are suitable for a multi-group path model, comparing variances and covariances across groups.

**Supplementary Table 5.** *Model Fit Indices for the Tests of Measurement Invariance between European Canadian and Japanese undergraduates for Latent Constructs (N_total_=442).*

| Model | $\boldsymbol{x}^{\boldsymbol{2}}$(*df*) | RMSEA  [90%CI] | CFI | TLI | SRMR | Model comparison (ΔCFI) |
| --- | --- | --- | --- | --- | --- | --- |
| *Independence* |  |  |  |  |  |  |
| Configural invariance | .000 (0) | .000 [.000, .000] | 1.000 | 1.000 | .000 |  |
| Metric invariance | **2.122 (2)** | **.024 [.000, .192]** | **.999** | **.998** | **.070** | **.001** |
| Scalar invariance | 11.813 (4) | .133 [.049, .224] | .952 | .928 | .095 | .047 |
| *Interdependence* |  |  |  |  |  |  |
| Configural invariance | .000 (0) | .000 [.000, .000] | 1.000 | 1.000 | .000 |  |
| Metric invariance | **.410 (2)** | **.000 [.000, .115]** | 1.000 | **1.027** | **.032** | **.000** |
| Scalar invariance | 14.241 (4) | .152 [.072, .241] | .943 | .915 | .088 | .057 |
| *Life satisfaction* |  |  |  |  |  |  |
| Configural invariance | 26.563 (10) | .122 [.067, .180] | .957 | .915 | .039 |  |
| Metric invariance | **32.513 (14)** | **.109 [.060, .159]** | **.952** | **.932** | **.071** | **.005** |
| Scalar invariance | 55.057 (18) | .136 [.096, .178] | .905 | .894 | .089 | .047 |

*Note.* Models showing data in bold have the best fit. RMSEA = root-mean-square error of approximation; CI = confidence interval; CFI = comparative fit index; TLI = Tucker&–Lewis index; SRMR = standardized root-mean-square residual.

References

Van de Vijver, F. J. R., & Leung, K. (1997). *Methods and data analysis for cross-cultural research.* Newbury Park, CA: SAGE.

**Relationships among Culture, Independence, Interpersonal Stress, and Life Satisfaction**

**Life Satisfaction.** The 5-item *Satisfaction with Life Scale* (Diener et al., 1985; e.g., “I am satisfied with my life”) was used to assess participants’ perceived life satisfaction on a scale from 1 (*strongly disagree)* to 7 (*strongly agree*). Internal consistencies for the measure of life satisfaction (European Canadian: *α* = .83; Japanese: *α* = .83) were good across cultures. We created the mean scores of life satisfaction, with higher scores signifying higher levels of life satisfaction.

**Path to life satisfaction.** We performed an exploratory serial mediation analysis (PROCESS Model 6) with independence and psychological symptoms from interpersonal situations as sequential mediators, linking culture and life satisfaction. There were three significant indirect effects: culture → independence → life satisfaction, indirect effect = −.253, 95%CI = [−.432, −.107]; culture → psychological symptoms from interpersonal situations → life satisfaction, indirect effect = −.232, 95%CI = [−.392, −.099]; and culture → independence → psychological symptoms from interpersonal situations → life satisfaction, indirect effect = −.035, 95%CI = [−.087, −.001]. These results indicated that compared to European Canadians, Japanese participants had lower levels of independence, *b* = −.710, *p* < .001, 95%CI = [−.907, −.514], which was associated with more psychological symptoms of stress arising from interpersonal situations, *b* = −.201, *p* = .051, 95%CI = [−.402, .000] (this path of the model, however, only approaches significance), which was linked to lower levels of life satisfaction, *b* = −.243, *p* < .001, 95%CI = [−.375, −.112] (Figure S1).

*
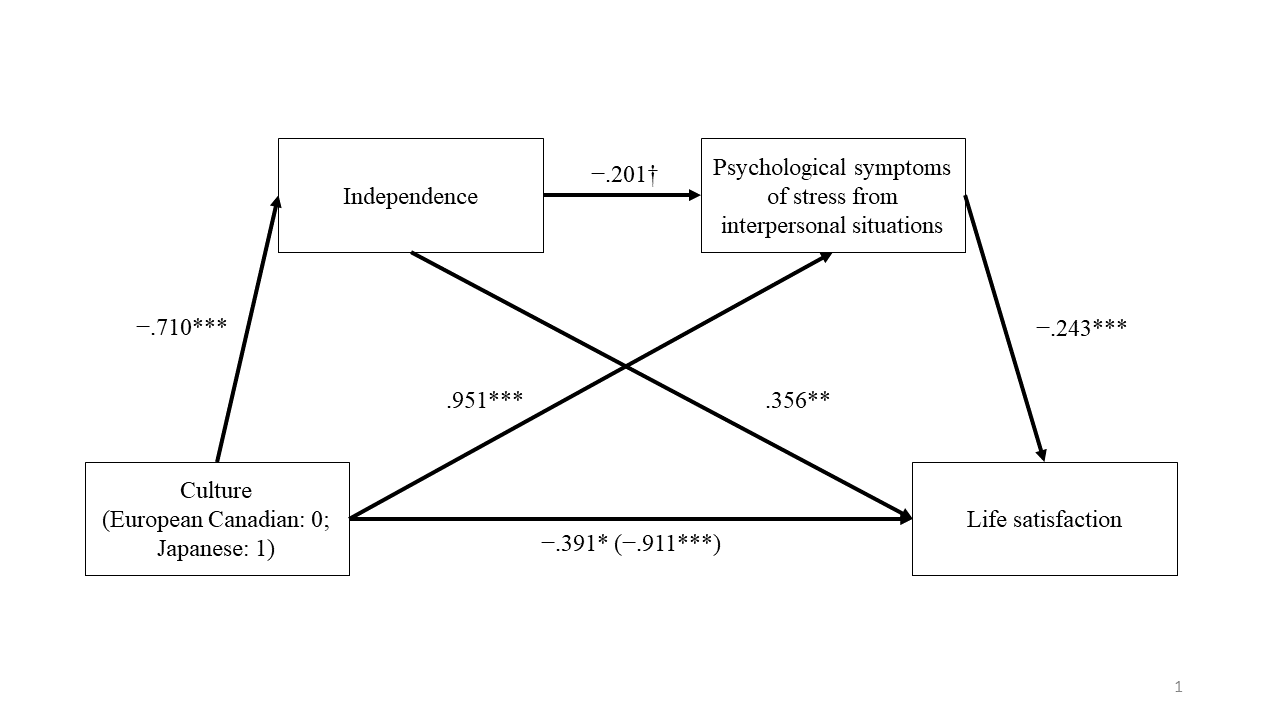
*

*Supplementary Figure 1.* Serial mediation analyses, Study 3.

*Note.* Unstandardized regression coefficients. Indirect effect from culture to life satisfaction via independence = −.253, 95% CI = [−.432, −.107]; indirect effect from culture to life satisfaction via psychological symptom of stress from interpersonal situations = −.232, 95% CI = [−.392, −.099]; indirect effect from culture to life satisfaction via independence and psychological symptom of stress from interpersonal situation = −.035, 95% CI = [−.087, −.001]. The number inside the brackets is the total effect (c), the number outside the brackets is the direct effect (c’).

†*p* = .051. ^*^*p* < .05. ^**^*p* < .01. ^***^*p* < .001 (two-tailed).

References

Diener, E. D., Emmons, R. A., Larsen, R. J., & Griffin, S. (1985). The Satisfaction with Life Scale. *Journal of Personality Assessment, 49*(1), 71-75. doi:10.1207/s15327752jpa4901_13
